# Transcriptomic Insights into Dual Temperature–Salinity Stress Response in “Shuike No. 1”, a Pioneering Rainbow Trout Strain Bred in China

**DOI:** 10.3390/biology14010049

**Published:** 2025-01-10

**Authors:** Xiaojun Liu, Gaochao Wang, Tianqing Huang, Enhui Liu, Wei Gu, Peng Fan, Kaibo Ge, Datian Li, Yunchao Sun, Gefeng Xu

**Affiliations:** 1 Heilongjiang River Fisheries Research Institute, Chinese Academy of Fishery Sciences, Harbin 150070, China; liuxiaojun6293@163.com (X.L.); gaochaowang@ymail.com (G.W.); huangtianqing@hrfri.ac.cn (T.H.); liuenhui@hrfri.ac.cn (E.L.); guwei@hrfri.ac.cn (W.G.); fanpeng@hrfri.ac.cn (P.F.); gekaibo@hrfri.ac.cn (K.G.); lidatian@hrfri.ac.cn (D.L.); sunyunchao@hrfri.ac.cn (Y.S.); 2 Key Laboratory of Freshwater Aquatic Biotechnology and Breeding, Ministry of Agriculture and Rural Affairs, Harbin 150070, China; 3 Engineering Technology Research Center for Cold Water Fish Breeding of Heilongjiang Province, Harbin 150070, China; 4 College of Fisheries and Life Sciences, Shanghai Ocean University, Shanghai 201306, China

**Keywords:** rainbow trout, salinity stress, temperature stress, RNA-seq, WGCNA

## Abstract

Global warming poses a significant threat to aquaculture, particularly for cold-water species like rainbow trout (*Oncorhynchus mykiss*). Understanding the molecular mechanisms underlying stress responses is crucial for developing resilient strains. This study investigates the dual stress of salinity and temperature response of “Shuike No. 1” (SK), a pioneering commercially bred rainbow trout strain in China, using RNA-sequencing of gill, intestine, and liver tissues from fish exposed to four treatment combinations: freshwater at 16 °C, freshwater at 25 °C, saltwater (30‰) at 16 °C, and saltwater at 25 °C. Differential gene expression analysis identified a substantial number of DEGs, with the liver showing the most pronounced response and a clear synergistic effect observed under combined high-temperature and salinity stress. Weighted gene co-expression network analysis (WGCNA) revealed stress-responsive gene modules and identified hub genes, primarily associated with gene expression, endoplasmic reticulum (ER) function, disease immunity, energy metabolism, and substance transport. Key hub genes included *klf9*, *fkbp5a*, *fkbp5b*, *ef2*, *cirbp*, *atp1b1*, *atp1b2*, *foxi3b*, *smoc1*, and *arf1*. Functional enrichment analysis confirmed the prominent role of ER stress, particularly the pathway “protein processing in the endoplasmic reticulum.” Our results reveal complex, tissue-specific responses to dual stress, with high temperature exerting a stronger influence than salinity. These findings provide valuable insights into the molecular mechanisms underpinning dual stress responses in SK, informing future breeding programs for enhanced resilience in the face of climate change.

## Simple Summary

Global warming, with its associated rising water temperatures and fluctuating salinity, poses a significant threat to global aquaculture, particularly for cold-water species like rainbow trout. Improving the tolerance of aquatic species to temperature is therefore essential. This study aims to investigate the molecular response mechanism of the new rainbow trout strain “Shuike No. 1” (SK) under the dual stress of temperature and salinity. Our data showed that the influence of high temperature on SK is greater than that of salinity, and the two have an obvious synergistic effect. Our findings provide valuable insights into the molecular mechanisms underpinning dual stress responses in SK, informing future breeding programs for enhanced resilience in the face of climate change.

## 1. Introduction

Global warming presents unprecedented challenges to global aquaculture, with rising water temperature, particularly affecting cold-water species that require specific environmental conditions for optimal growth and survival [1,2,3]. Among these species, rainbow trout (*Oncorhynchus mykiss*) holds significant economic importance in global aquaculture, valued for its high nutritional content, superior flesh quality, and absence of intermuscular bones [4,5,6]. Originally native to North American waters [6], rainbow trout have been successfully introduced worldwide, with major production centers in Europe and Asia [5,7,8]. The global rainbow trout production has shown steady growth over the past decade, reaching approximately 960,000 tons in 2020, with inland aquaculture contributing 740,000 tons and marine farming accounting for 220,000 tons [4]. This substantial production reflects the species’ importance in meeting global seafood demand.

Rainbow trout exists in two distinct morphotypes, a freshwater resident form and an anadromous form known as steelhead, which naturally migrates between freshwater and marine environments [9]. This species exhibits specific environmental requirements for optimal growth and survival, thriving in temperatures between 12 and 18 °C [10,11,12], with a broader tolerance range of 8–22 °C [13]. Temperatures beyond 25 °C or below 5 °C can be lethal [14,15]. While rainbow trout is traditionally cultivated in freshwater systems, China faces significant constraints in inland aquaculture expansion due to limited cold freshwater resources. The species’ natural euryhaline capability, demonstrated by steelhead populations, has already been successfully exploited in marine aquaculture globally. Following the success of marine rainbow trout farming in countries like Norway [5] and Denmark [16], China has begun exploring sea-cage farming as a promising strategy to overcome its cold freshwater limitations [17].

However, global warming poses a significant threat to rainbow trout marine farming through rising water temperatures. Temperature stress significantly impacts fish physiology, affecting osmoregulation, metabolism, immune response, and reproductive capacity [18,19,20]. High temperature acts as a master abiotic factor, influencing virtually all physiological processes and behaviors in fish [19]. The interaction between thermal and salinity stress can be particularly devastating, as elevated temperatures often compromise fish osmoregulatory capacity, thereby reducing salinity tolerance [18,21,22].

The physiological response to dual temperature–salinity stress involves complex interactions across multiple organ systems, with the gills, intestines, and liver forming an integrated network crucial for maintaining homeostasis. The gills, as the primary interface between the fish and its aquatic environment, play a paramount role in both osmoregulation and respiratory gas exchange [23]. It features specialized mitochondria-rich cells (MRC), also known as chloride cells (CC), which regulate ion balance [24,25]. The intestine serves dual functions in osmoregulation and nutrient processing. In response to salinity stress, it modulates water absorption and ion transport through specialized transporters and channels, while simultaneously maintaining its role in nutrient uptake and immune defense [26,27]. The intestinal epithelium also acts as a crucial barrier, with tight junctions regulating paracellular permeability and protecting against pathogen invasion [28]. The liver, functioning as the central metabolic hub, coordinates the stress response by regulating glucose metabolism, synthesizing stress proteins, and managing energy allocation [29]. During periods of environmental stress, it upregulates metabolic pathways to meet increased energy demands, produces various stress-response proteins, and maintains xenobiotic biotransformation capabilities [29].

“Shuike No. 1” (SK), China’s first independently bred rainbow trout strain (breed registration number GS-01-001-2021), exhibits superior traits including enhanced growth rate and improved feed conversion efficiency. However, as China expands rainbow trout production from traditional cold-water regions to marine environments, SK’s performance under the combined challenges of elevated temperature and salinity remains poorly characterized, particularly at the molecular level. Understanding the physiological and molecular mechanisms underlying SK’s response to these dual stressors is crucial for successful marine farming, especially given the challenges posed by global warming.

Recent advances in high-throughput sequencing technologies and bioinformatics have enabled comprehensive analysis of transcriptional responses to environmental stressors. Previous studies have employed these approaches to investigate stress responses in various aquaculture species, including Atlantic salmon (*Salmo salar*) [21], large yellow croaker (*Larimichthys crocea*) [30], and olive flounder (*Paralichthys olivaceus*) [31]. This study aims to elucidate the molecular responses of SK rainbow trout to concurrent high temperature and salinity stress through comprehensive transcriptomic analysis of gill, intestine, and liver tissues. By employing RNA-seq technology and advanced bioinformatic analyses, we seek to identify key genes, pathways, and regulatory networks involved in stress responses. These findings will provide crucial insights for developing more resilient strains and optimizing aquaculture practices in the face of climate change.

## 2. Materials and Methods

### 2.1. Experimental Design and Sample Collection

This study utilized 60 healthy rainbow trout (“Shuike No. 1”) individuals, sourced from the Bohai Experimental Station of the Heilongjiang River Fisheries Research Institute (Harbin, China), marked with PITs (Passive Integrated Transponders) for identification and tracking. Half a month later, they were transported to our experimental site, with an average body length of 32.08 ± 2.10 cm and body weight of 392.31 ± 33.75 g. After a one-week acclimation period in a temperature-controlled aquarium (180 cm × 60 cm × 50 cm) maintained at 16 ± 0.2 °C, pH 7.1–7.5, and 7.0 ± 0.5 mg/L dissolved oxygen, the fish were randomly assigned to four treatment groups (*n* = 15 per group, and one group per aquarium): freshwater at 16 °C (Group F), freshwater at 25 °C (Group T), seawater at 16 °C (Group S), and seawater at 25 °C (Group D). In Group F, fish were maintained at the breeding conditions throughout the acclimation period (water temperature: 16 ± 0.2 °C, pH: 7.1–7.5, dissolved oxygen: 7.0 ± 0.5 mg/L), and samples were collected after seven days. In Group T, the temperature was gradually increased at a rate of 1 °C/day until reaching 25 °C, and samples were collected after three days at 25 °C. In Group S, the salinity was gradually increased from freshwater to 30‰ at a rate of 4‰/day, and samples were collected after seven days at 30‰ salinity. In Group D, salinity was increased as in Group S. Thereafter, the temperature was raised as in Group T to 25 °C. Samples were collected after three days at 25 °C and 30‰ salinity.

Water quality parameters (temperature, pH, dissolved oxygen) were monitored daily and maintained within specified ranges (±0.5‰ for salinity, ±0.2 °C for temperature). All groups were subjected to the natural light cycle, and fish were fed commercial pellet feed twice daily; each dose was 1% of body weight during the acclimation period. After the respective treatment periods, six individuals from each group were euthanized using MS-222, and their gills, intestines, and livers were dissected immediately on an ice tray. Tissues were quickly frozen in liquid nitrogen and subsequently stored in an ultra-low temperature refrigerator at −80 °C for RNA extraction.

### 2.2. RNA Extraction and Sequencing

Total RNA was extracted from three tissues (gill, intestine, and liver) using TRIzol reagent (Invitrogen, Carlsbad, CA, USA) following the manufacturer’s protocol. Genomic DNA contamination was removed using RNase-free DNase I (TaKaRa, Shiga, Japan). RNA concentration and integrity were assessed using 1% agarose gel electrophoresis and a NanoDrop ND-1000 spectrophotometer (NanoDrop Technologies Inc., Wilmington, NC, USA). The quality of RNA samples was further evaluated using an Agilent 2100 Bioanalyzer (Agilent Technologies, Santa Clara, CA, USA). A total of 72 sequencing libraries were constructed (three tissues × six biological replicates × four treatments). The libraries were sequenced on the Illumina NovaSeq 6000 platform using a paired-end (PE) 150 sequencing strategy. Sequencing samples were named according to their treatment group, tissue, and replicate number. For example, in Group F, the gill samples were labeled FG1-FG6, the intestine samples were FI1-FI6, and the liver samples were FL1-FL6. A similar naming convention was applied to the samples from groups S, T, and D.

### 2.3. Data Processing and Differential Gene Expression Analysis

Raw sequencing reads were first processed using fastp [32] (v0.23.4) to remove residual adapter sequences and perform quality filtering. The following parameters were used: -q 20 -u 50 -n 15 -l 150, with other parameters set to default values. High-quality clean reads were then aligned to the rainbow trout reference genome (NCBI assembly number GCF_013265735.2) using Hisat2 [33] (v2.2.1) with default parameters. Transcript assembly was performed using StringTie [34] (v2.2.1). Gene counts and FPKM values were calculated using python (v3.10.6) and the prepDE.py of StringTie (v2.2.1). Differential gene expression analysis was conducted using Group F as the control group. Comparisons were performed between the control group and the following treatment groups: Group S, Group T, and Group D. Differential expression analysis was conducted separately for each tissue: gill (SG vs. FG, TG vs. FG, DG vs. FG), intestine (SI vs. FI, TI vs. FI, DI vs. FI), and liver (SL vs. FL, TL vs. FL, DL vs. FL). Significant differentially expressed genes (DEGs) were identified using DESeq2 [35] R package (v1.44.0). DEGs were defined as those with |log_2_FoldChange| ≥ 1 and *p*.adjust (adjusted *p*-value) < 0.05.

### 2.4. Weighted Gene Co-Expression Network Analysis (WGCNA)

To construct a robust and informative gene co-expression network, a subset of genes with the highest variation across tissues was selected for weighted gene co-expression network analysis (WGCNA) using the WGCNA [36] R package (v1.69). The top 5000 genes with the largest absolute deviations in median expression across the three tissues (gill, intestine, liver) were chosen to minimize background noise and enhance network construction accuracy. Gene expression correlation coefficients were calculated using Pearson correlation. Suitable soft threshold powers for scale-free topological models were determined for each tissue (gill: eight, intestine: eleven, liver: seven) based on a model fit index (R^2^ ≥ 0.85) and a soft threshold power ≤ 15 (Figure S1).

Genes were subsequently clustered into modules based on their expression patterns, with the “grey” module excluded in the subsequent analysis as it was considered invalid. The module membership (MM) of each gene was calculated as the absolute value of the Pearson correlation coefficient between its expression pattern and the corresponding feature gene for that module. Gene significance (GS) was defined as the absolute value of the association between gene expression and a specific trait. Candidate hub genes within target modules were identified by setting thresholds for both MM and GS.

A candidate gene interaction network was constructed based on correlations among intramodular genes and visualized using Cytoscape [37] (v3.10.2) software. These candidate genes were then mapped with DEGs and the top 5%, 10%, and 30% of intramodular connectivity genes (based on module size) to identify hub genes associated with the dual stress of high salinity and temperature.

### 2.5. Functional Enrichment Analysis

Annotation information for rainbow trout genes was retrieved using the AnnotationHub [38] R package (v3.12.0) with the OrgDb accession number AH114235. Gene Ontology (GO) and Kyoto Encyclopedia of Genes and Genomes (KEGG) enrichment analyses were performed on differentially expressed genes (DEGs) and genes within target modules using the clusterProfiler [39] R package (v4.12.0). Enriched GO terms and KEGG pathways were ranked by adjusted *p*-value (*p*.adjust < 0.05). KEGG pathways with significant enrichment of DEGs across the three tissues under dual stress were visualized using the Pathview [40] R package (v1.44.0). Finally, GO terms and KEGG pathways enriched for hub genes were identified, and hub genes were categorized based on their reported primary functions.

### 2.6. Validation of RNA-seq Data by qRT-PCR

To validate the accuracy and reliability of the RNA-seq data, 12 differentially expressed genes (DEGs) were randomly selected for quantitative real-time polymerase chain reaction (qRT-PCR) analysis. Gene-specific primers were designed using Primer Premier 5.0 software based on the nucleotide sequences of these genes, with *β-actin* selected as the internal reference gene (Table S1). qRT-PCR was performed using the 2 × S6 Universal SYBR qPCR Mix (EnzyArtisan, Shanghai, China) according to the manufacturer’s protocol. Each cDNA sample was run in triplicate. Relative expression levels of genes were calculated using the 2^−ΔΔCt^ method, and the results were visualized using the ggplot2 (v3.5.1) R package.

## 3. Results

### 3.1. RNA Sequencing Data Quality Assessment

A total of 72 RNA-seq datasets, comprising 2,995,964,960 raw reads, were obtained from the gill, intestine, and liver tissues of SK rainbow trout subjected to four different rearing conditions (F, S, T, and D). Following quality control using fastp (v0.23.4), 2,872,953,930 clean reads were retained, exhibiting high quality with Q20 values ranging from 98.12% to 98.62%, Q30 values ranging from 94.43% to 96.08%, and GC content ranging from 47.74% to 50.73%. Importantly, 86.68% to 93.62% of the clean reads were successfully mapped to the rainbow trout reference genome (GCF_013265735.2), with an average mapping rate of 90.65% (Table S2). These results indicate high-quality RNA-seq data suitable for downstream analysis.

### 3.2. Differential Gene Expression Analysis Reveals Tissue-Specific Responses to Dual Stress

Differential gene expression analysis was performed to identify genes whose expression levels differed significantly between the various treatment groups. Pairwise comparisons were made between the control group F and each of the experimental groups (S, T, and D).

**Figure 1:**
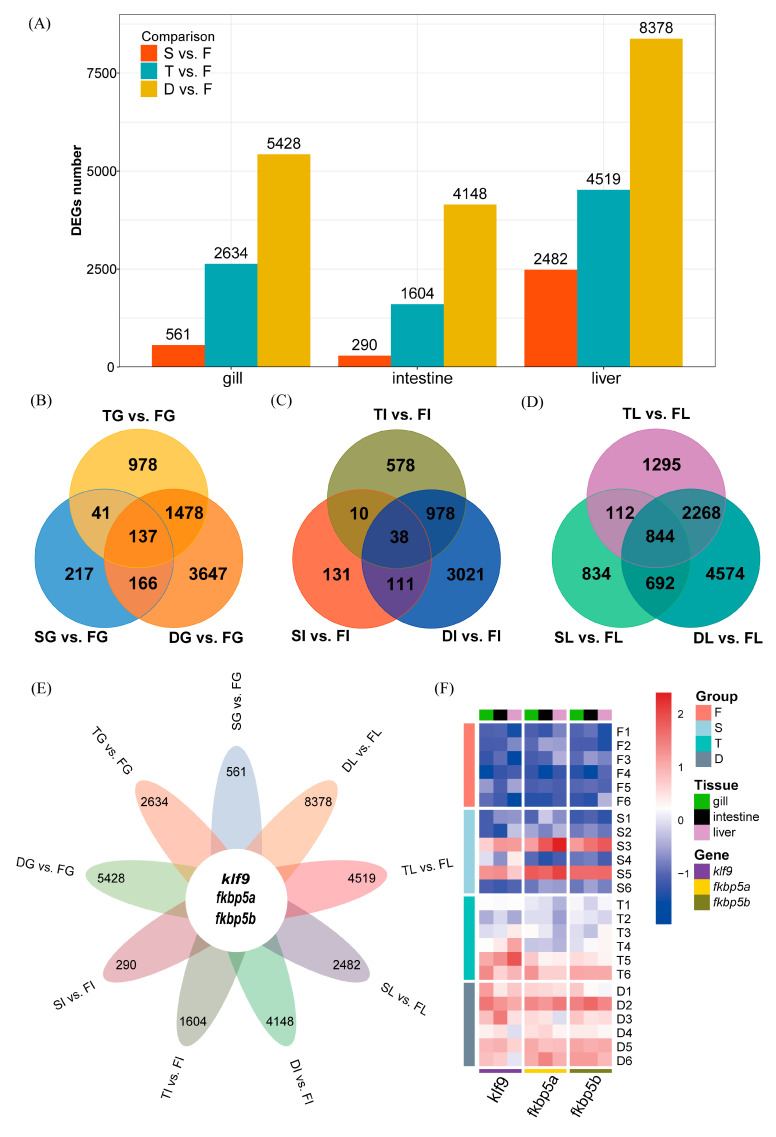
Differential gene expression analysis in response to dual salinity and temperature stress in rainbow trout “Shuike No. 1” (SK). (**A**) Number of differentially expressed genes (DEGs) in the gills, intestines, and liver of SK under high salinity (S vs. F), high temperature (T vs. F), and dual stress (D vs. F) conditions. (**B**–**D**) Venn diagrams illustrating the overlap of DEGs in the gills (**B**), intestines (**C**), and liver (**D**) under the three stress conditions. (**E**) Petal diagram showing DEGs shared across all three tissues and stress conditions. Three common DEGs (*klf9*, *fkbp5a*, and *fkbp5b*) are highlighted in the center of the diagram. (**F**) Heatmap displaying the expression levels of the three common DEGs (*klf9*, *fkbp5a*, and *fkbp5b*) in different tissues under four treatment conditions (F, S, T, and D).

A substantial number of differentially expressed genes (DEGs) were identified in all three tissues (gill, intestine, and liver) (Figure 1A and Table S3). The number of DEGs was generally lowest in the S vs. F comparison and highest in the D vs. F comparison. Furthermore, the combined number of DEGs in S vs. F and T vs. F was significantly lower than the number observed in D vs. F, suggesting a more pronounced impact of dual stress (D vs. F) on gene expression.

Venn diagrams were used to illustrate the overlap of DEGs across the different stress conditions (Figure 1B–D). In the gills, a significant number of DEGs were shared between T vs. F and D vs. F, indicating a similar response to high temperature in both freshwater and seawater. The liver showed the highest degree of overlap across all three comparisons, suggesting a more pronounced and complex response to the dual stress.

A closer examination of the shared DEGs across all three tissues revealed three common genes: *klf9* and two peptidyl–prolyl cis–trans isomerase genes (*fkbp5a* and *fkbp5b*) (Figure 1E and Table S4). *Klf9*, an important transcription factor known to regulate gene transcription and potentially involved in immune response, showed significantly lower expression in groups F and S compared to groups T and D (with the exception of samples S3 and S5), suggesting a potential role in the response to high temperature (Figure 1F). *Fkbp5*, a gene involved in proper protein folding, also displayed similar expression patterns, with reduced expression in groups F and S. Further investigation into the functions of these genes and their potential regulatory roles in the stress response pathways is warranted.

### 3.3. Functional Enrichment Analysis of Differentially Expressed Genes

To further investigate the biological processes and pathways affected by the combined stress of high salinity and high temperature, GO and KEGG enrichment analyses were performed specifically on the DEGs identified in the D vs. F comparison.

**Figure 2:**
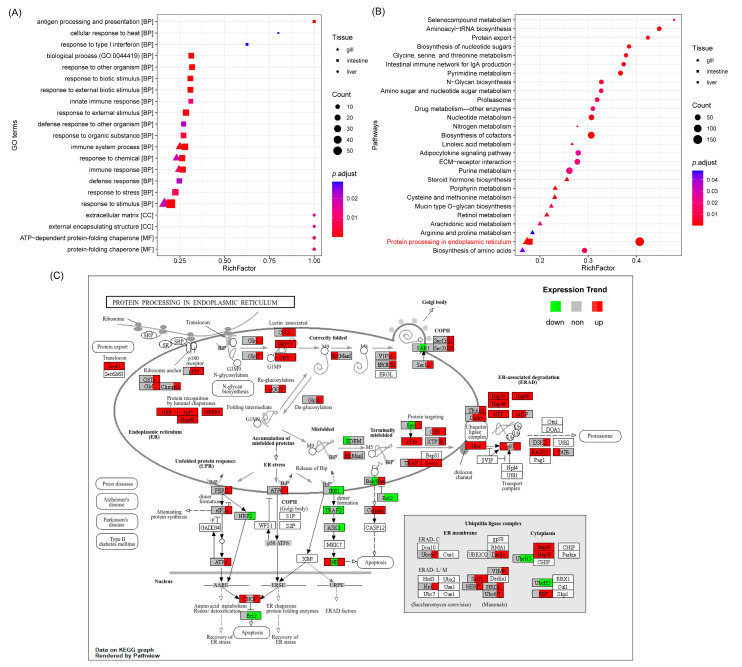
Functional enrichment analysis of differentially expressed genes (DEGs) in response to dual salinity and temperature stress in rainbow trout (SK). (**A**) Enriched GO terms in the gills, intestines, and liver of SK under the dual stress condition (D vs. F). GO terms are categorized by their hierarchical level (“biological process,” “cellular component,” and “molecular function”). (**B**) Significantly enriched KEGG pathways (*p*.adjust < 0.05) in the gills, intestines, and liver under the dual stress condition. The “protein processing in the endoplasmic reticulum” pathway (ko04141), which is shared across all three tissues, is highlighted in red. (**C**) KEGG pathway map for protein processing in the endoplasmic reticulum (ko04141), illustrating the expression patterns of DEGs in the gills, intestines, and liver. Genes are color-coded as follows: Green, Downregulated expression; Red, Up-regulated expression; Grey, Not differentially expressed; Blank, Gene is not differentially expressed in any tissue or is not associated with rainbow trout.

GO enrichment analysis revealed tissue-specific patterns of enriched terms (Figure 2A). In the gills and intestines, DEGs were primarily enriched in terms related to “biological process,” while in the liver, enrichment was observed for terms related to “cellular component” and “molecular function.” Notably, the liver displayed significant enrichment for terms such as extracellular matrix (GO:0031012), external encapsulating structure (GO:0030312), protein-folding chaperone (GO:0044183), and ATP-dependent protein-folding chaperone (GO:0140662). In contrast, DEGs in the gills and intestines were commonly enriched for terms associated with responses to a stimulus (GO:0050896), responses to a chemical (GO:0042221), immune system processes (GO:0002376), and immune responses (GO:0006955).

KEGG pathway enrichment analysis identified significantly enriched pathways (*p*.adjust < 0.05) in each tissue (Figure 2B). In the gills, pathways related to cysteine and methionine metabolism (ko00270), retinol metabolism (ko00830), and protein processing in the endoplasmic reticulum (ko04141) were significantly enriched. In the intestines, only protein processing in the endoplasmic reticulum (ko04141) was significantly enriched. The liver exhibited significant enrichment in pathways related to protein processing in the endoplasmic reticulum (ko04141), biosynthesis of cofactors (ko01240), and nucleotide metabolism (ko01250).

The consistent enrichment of the “protein processing in the endoplasmic reticulum” pathway across all three tissues (Figure 2C) highlights the potential role of endoplasmic reticulum (ER) stress in response to dual stress. This pathway was most active in the liver, followed by the gills. Notably, genes associated with protein chaperone recognition (*nef*, *bip*, *grp94*, and *hsp40*) were up-regulated in all three tissues. Genes involved in protein-folding (*crt*, *cnx*, *pdis*, *erp57*, etc.) were up-regulated in at least one tissue, and the majority of genes involved in protein export (*ergic53*, *vip36*, *sec12*, *sec23/24*, *sec13/31*, and *sec61*) as well as some genes involved in apoptosis were up-regulated in the liver. Genes within the endoplasmic reticulum-associated degradation (ERAD) mechanism were also predominantly up-regulated across all tissues.

### 3.4. Identification of Gene Modules and Hub Genes in the Gills Associated with Dual Stress

**Figure 3:**
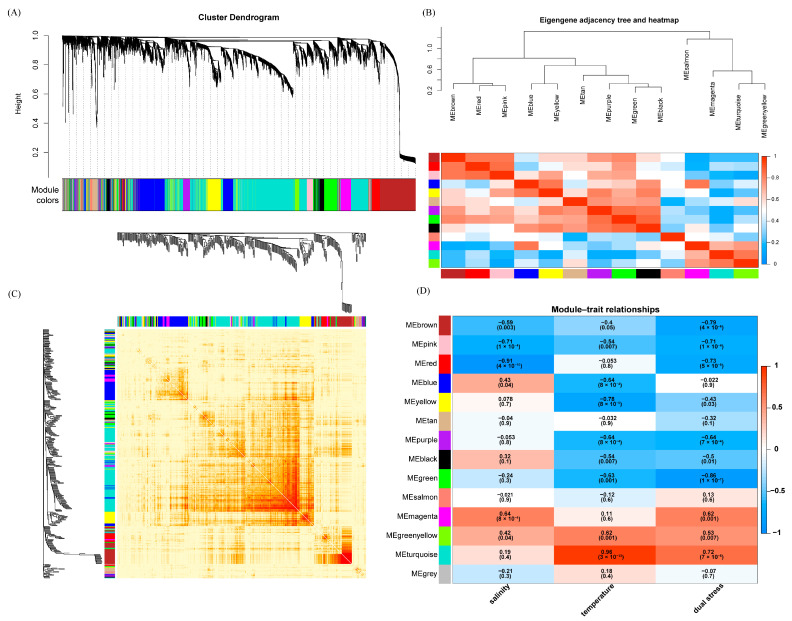
Identification of gene modules associated with dual salinity and temperature stress in the gills. (**A**) Cluster dendrogram of the top 5000 most variable genes in the gill tissue, as identified from 18 RNA-seq datasets. These genes were clustered into 14 distinct modules, color-coded for identification. (**B**) Eigengene adjacency tree and heatmap, illustrating the hierarchical clustering of modules based on their eigengene adjacency, revealing distinct expression patterns. (**C**) Topological Overlap Matrix (TOM) plot. Four hundred genes were randomly selected from the 5000 most variable genes for visualization. Red color intensity represents the degree of topological overlap between genes (deeper red indicates a higher overlap). (**D**) Heatmap depicting the correlations between module eigengenes and high salinity and/or high temperature. Negative and positive correlations are indicated in blue and red, respectively.

Weighted gene co-expression network analysis (WGCNA) was performed to identify gene modules associated with dual stress response in the gill tissue of SK rainbow trout. A total of 14 distinct modules, with gene numbers ranging from 45 (salmon module) to 1781 (turquoise module), were identified based on gene co-expression patterns (Figure 3A and Table S5). These modules were further clustered based on their eigengene adjacency, revealing distinct expression patterns (Figure 3B). A Topological Overlap Matrix (TOM) plot demonstrated a low level of topological overlap and a high degree of scale-freeness among the 14 modules, suggesting a robust network structure (Figure 3C).

Correlation analysis between module eigengenes and stress-related traits identified modules strongly associated with salinity or temperature (Figure 3D). The pink, red, and magenta modules were highly correlated with salinity, while the blue, yellow, purple, green, green-yellow, and turquoise modules exhibited a strong association with temperature.

The brown, pink, red, purple, green, magenta, and turquoise modules showed a significant correlation with the combined stress of high salinity and high temperature, with the turquoise and green modules displaying the highest positive and negative correlations, respectively (R^2^ = 0.72 and R^2^ = −0.86). GO and KEGG enrichment analyses were performed on the genes within the turquoise and green modules to uncover their associated biological functions (Figure S2). These analyses revealed significant enrichment in GO terms and KEGG pathways linked to substance transport, protein processing, energy metabolism, and disease immunity. For example, the GO terms electron transport chain (GO:0022900), protein-folding chaperone (GO:0044183), NADH dehydrogenase activity (GO:0003954), envelope (GO:0031975), the KEGG pathway phagosome (ko04145), protein processing in the endoplasmic reticulum (ko04141), and oxidative phosphorylation (ko00190).

**Figure 4:**
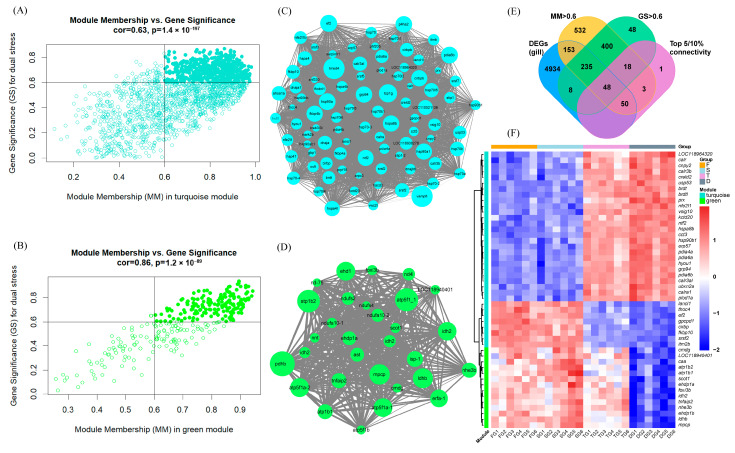
Identifying hub genes associated with dual stress in the gills. (**A**,**B**) Scatter plots of gene significance (GS) and module membership (MM) for the turquoise (**A**) and green (**B**) modules. Genes with GS > 0.6 and MM > 0.6 (indicated by horizontal lines) are potential hub genes, highlighted in darker colors. (**C**,**D**) Co-expression networks of candidate hub genes in the turquoise (**C**) and green (**D**) modules, constructed based on intramodular connectivity. Circle size is proportional to the intramodular connectivity. Genes with connectivity greater than 275.38 (**C**) and 39.72 (**D**) are potential hub genes. (**E**) Venn diagram illustrating the overlap of candidate hub genes identified in the turquoise and green modules. (**F**) Clustering heatmap of hub gene expression in the turquoise and green modules. Expression levels were normalized using log_2_ (FPKM + 1).

To identify hub genes within these modules, a combination of gene significance (GS), module membership (MM), intramodular connectivity, and DEGs was utilized. Using thresholds of GS > 0.6 and MM > 0.6, 532 and 169 candidate hub genes were identified within the turquoise and green modules, respectively (Figure 4A,B). Additionally, co-expression networks were constructed for the turquoise and green modules based on intramodular connectivity, with genes in the top 5% and 10% (with connectivity values above 275.38 and 39.72, respectively) identified as candidate hub genes (Figure 4C,D). Finally, these candidate hub genes were intersected with the DEGs identified in the D vs. F comparison in the gill tissue, yielding a total of 48 hub genes associated with the dual stress response in the gill of SK (Figure 4E and Table S6). A heatmap of these hub genes showed strong correlations between the turquoise module and high temperature, while the green module exhibited a closer association with the combined effects of high salinity and temperature (Figure 4F). Specifically, genes like *cct3*, *ef2*, and *cirbp* were closely related to high temperature, whereas *atp1b1*, *atp1b2*, *mpcp*, and *foxi3b* showed a stronger association with the dual stress.

### 3.5. Identification of Gene Modules and Hub Genes in the Intestines Associated with Dual Stress

WGCNA was applied to the intestinal tissue of SK rainbow trout to identify gene modules associated with the dual stress response. A total of 15 distinct modules, with gene numbers ranging from 71 (cyan module) to 1483 (turquoise module), were identified based on gene co-expression patterns (Figure S3A and Table S5). These modules were further clustered based on their eigengene adjacency, revealing divergent expression profiles (Figure S3B). A TOM plot demonstrated a high degree of scale-free topology among the 15 modules, suggesting a robust network structure (Figure S3C).

Correlation analysis between module eigengenes and stress-related traits revealed that the black module exhibited the most positive correlation with the combined stress (R^2^ = 0.66). In contrast, the pink and green-yellow modules displayed the highest negative correlations (R^2^ = −0.46) (Figure S3D). GO and KEGG enrichment analyses were performed on the genes within the black, pink, and green-yellow modules to uncover their associated biological functions (Figure S2). These analyses revealed significant enrichment in GO terms and KEGG pathways linked to substance transport, protein processing, energy metabolism, and disease immunity, such as the GO terms electron transport chain (GO:0022900), envelope (GO:0031975), protein-folding chaperone (GO:0044183), NADH dehydrogenase activity (GO:0003954), the KEGG pathway proteasome (ko03050), and oxidative phosphorylation (ko00190).

To identify hub genes within these modules, a combination of GS, MM, intramodular connectivity, and DEGs was utilized. Using thresholds of GS > 0.5 and MM > 0.5, a total of 41, 11, and 13 candidate hub genes were identified within the black, pink, and green-yellow modules, respectively (Figure S4A–C). Additionally, co-expression networks were constructed for the black, pink, and green-yellow modules based on intramodular connectivity, with genes in the top 30% (with connectivity values above 43.03, 141.33, and 26.05, respectively) identified as candidate hub genes (Figure S4D–F). Finally, these candidate hub genes were intersected with the DEGs identified in the D vs. F comparison in the intestinal tissue, yielding a total of 12 hub genes associated with the dual stress response in the intestine of SK (Figure S4G and Table S6). A heatmap of these hub genes showed that genes like *ckba*, *smoc1*, and *dpm2* were more closely associated with high temperatures (Figure S4H).

### 3.6. Identification of Gene Modules and Hub Genes in the Liver Associated with Dual Stress

WGCNA was applied to the liver tissue of SK rainbow trout to identify gene modules associated with the dual stress response. A total of nine distinct modules, with gene numbers ranging from 43 (pink module) to 1386 (turquoise module), were identified based on gene co-expression patterns (Figure S5A and Table S5). These modules were further clustered based on their eigengene adjacency, revealing divergent expression profiles (Figure S5B). A TOM plot demonstrated a high degree of independence among the nine modules, suggesting a robust network structure (Figure S5C).

Correlation analysis between module eigengenes and stress-related traits revealed that the brown and turquoise modules exhibited the highest positive correlations with the combined stress (R^2^ = 0.47 and R^2^ = 0.48, respectively). In contrast, the yellow module displayed the highest negative correlation (R^2^ = −0.87) (Figure S5D). GO and KEGG enrichment analyses were likewise performed on the genes within the brown, turquoise, and yellow modules to uncover their associated biological functions, and the results were similar to gill and intestinal tissues (Figure S2).

To identify hub genes within these modules, a combination of GS, MM, intramodular connectivity, and DEGs was utilized. Using thresholds of GS > 0.6 and MM > 0.6, a total of 65, 109, and 528 candidate hub genes were identified within the brown, turquoise, and yellow modules, respectively (Figure S6A–C). Additionally, co-expression networks were constructed for the brown, turquoise, and yellow modules based on intramodular connectivity, with genes in the top 5% (with connectivity values above 168.51, 307.88, and 344.03, respectively) identified as candidate hub genes (Figure S6D–F). Finally, these candidate hub genes were intersected with the DEGs identified in the D vs. F comparison in the liver tissue, yielding a total of 46 hub genes associated with the dual stress response in the liver of SK (Figure S6G and Table S6). A heatmap of these hub genes showed that genes like *golga4* were more closely associated with high temperature, while *arf1* and *copa* displayed a stronger association with the dual stress (Figure S6H).

### 3.7. Functional Analysis of Hub Genes in the Gills, Intestines, and Liver

**Figure 5:**
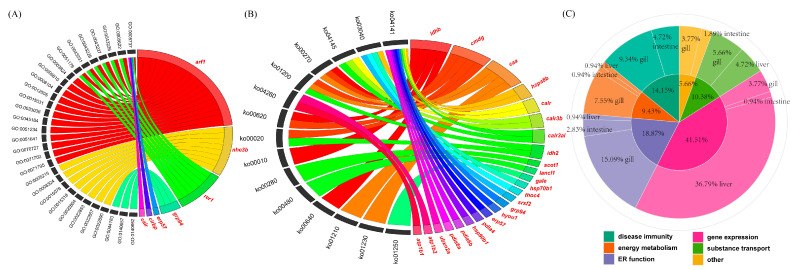
Functional analysis of hub genes in the gills, intestines, and liver. (**A**) Chord diagram illustrating the relationships between hub genes and enriched GO terms (biological process). Hub genes are highlighted in red. (**B**) Chord diagram showing the relationships between hub genes and enriched KEGG pathways. Hub genes are highlighted in red. (**C**) Double-layer pie chart depicting the functional roles and tissue distribution of hub genes. The inner layer, represented in darker colors, illustrates the primary functional roles of hub genes. The outer layer, shown in lighter colors, indicates the tissue of origin for the hub genes.

To gain further insights into the roles of hub genes under the dual stress of high salinity and temperature, the results of all functional enrichment analyses were reviewed. As depicted in Figure 5A,B, a subset of hub genes was enriched in specific GO terms and KEGG pathways, with detailed information provided in Table S7. Notably, more hub genes were found in the cytoplasm (GO:005737), protein processing in the endoplasmic reticulum (ko04141), and spliceosome (ko03040). The genes *arf1*, *nhe3b*, *rer1*, *ldhb*, *cmdg*, and *caa* appeared in more GO terms or KEGG pathways. In addition, based on their reported main functional roles, these hub genes were categorized into groups associated with gene expression, ER function, disease immunity, energy metabolism, and substance transport (Figure 5C).

### 3.8. Validation of RNA-seq Data by qRT-PCR

To validate the accuracy and reliability of the RNA-seq datasets, a subset of 12 DEGs was randomly selected for quantitative real-time PCR (qRT-PCR) analysis. The selected genes included *fkbp5a*, *fkbp5b*, *klf9*, *calr*, *plod1a*, *hyou1*, *hspa8b*, *grp94*, *smoc1*, *golga4*, *rer1*, and *stx5*. As shown in Figure S7, the qRT-PCR and RNA-seq expression patterns for these genes were highly congruent across various treatments in the gills, intestines, and liver of rainbow trout. These findings provide compelling evidence for the accuracy and reliability of the RNA-seq datasets used in this study.

## 4. Discussion

### 4.1. Differential Gene Expression and Synergistic Stress Effects

Our differential gene expression analysis unequivocally demonstrates a synergistic effect of combined high salinity and high temperature stress on SK. The substantially higher number of DEGs observed under dual stress (D vs. F; gill: 5428, intestine: 4148, liver: 8378) compared to single stressors alone (S vs. F: gill: 561, intestine: 290, liver: 2482; T vs. F: gill: 2634, intestine: 1604, liver: 4519) indicates that the combined effects are not merely additive but rather involve complex interactions between stress response pathways. This reinforces the importance of studying combined stressors in a realistic context, as they reflect the actual environmental challenges faced by fish in a changing climate. Importantly, SK exhibited a relatively muted response to high salinity alone, with a lower number of DEGs in the S vs. F comparison. This suggests a pre-existing adaptation to salinity changes, which aligns with the euryhaline capacity observed in anadromous steelhead [9], Atlantic salmon [21], and other related salmonid species. Specifically, this inherent salinity tolerance could be attributed to the efficient osmoregulatory mechanisms present in the gill, intestine, and other osmoregulatory tissues, allowing them to maintain ionic balance even in fluctuating salinity environments.

### 4.2. Key Genes and Pathways in Dual Stress Response

The consistent upregulation of *klf9*, *fkbp5a*, and *fkbp5b* across all tissues under dual stress points to their central roles in coordinating the stress response. *klf9*, a transcription factor in the SP/KLF family, acts as a feedforward regulator in the glucocorticoid receptor (GR) signaling pathway [41,42], a central player in the vertebrate stress response. *klf9* has been implicated in diverse physiological processes, including hematopoiesis, T-lymphocyte production [43], and the regulation of oxygen consumption [41]. Its upregulation in SK likely reflects its involvement in orchestrating the complex interplay of immune modulation, metabolic adjustment, and oxygen consumption required to cope with dual stress. The increased expression of *fkbp5a* and *fkbp5b*, peptidyl–prolyl cis–trans isomerases with known roles in protein folding [44] and GR regulation [41], further supports the importance of GR signaling in the dual stress response. These proteins interact with Hsp90, modulating GR activity and potentially influencing downstream stress-responsive genes [44,45,46]. Additionally, *fkbp5* is also involved in disease immunity, and its overexpression will increase the risk of contracting diseases [47,48]. However, the observation that some SK individuals displayed elevated co-expression of these genes even under seawater conditions may suggest an inability of them to adapt to a high-salinity osmotic environment. Furthermore, landlocked rainbow trout live in freshwater for a long time, which potentially leads to a loss or weakening of salinity adaptation capabilities in certain individuals. Consequently, there may be underlying genetic variations associated with tolerance within the SK population. These variations could be exploited in selective breeding programs, using these genes as potential markers for identifying and propagating stress-tolerant individuals. The roles of *klf9* and *fkbp5* in stress response has also been documented in other fish species, such as zebrafish (*Danio rerio*) [41,43,49,50], orange-spotted grouper (*Epinephelus bleekeri*) [51], grass carp (*Ctenopharyngodon idellus*) [47], tilapia (*Oreochromis niloticus*) [48], and rare minnows (*Gobiocypris rarus*) [52].

### 4.3. WGCNA Reveals Tissue-Specific Stress-Responsive Modules

To understand the broader regulatory context of these key genes, we performed WGCNA, identifying modules of co-expressed genes associated with dual stress in each tissue. In our study, WGCNA revealed distinct stress-responsive modules in each tissue, highlighting the tissue-specific nature of the dual stress response. In the gills, the turquoise and green modules emerged as highly correlated with high temperature and dual stress, respectively. These modules contain hub genes such as *cct3*, *ef2*, and *cirbp* (turquoise module, associated with high temperature), as well as *atp1b1*, *atp1b2*, *mpcp*, and *foxi3b* (green module, associated with dual stress). These hub genes are likely involved in coordinating the transcriptomic responses in the gills. The functional roles of these genes, chaperone activity, protein synthesis, RNA binding, ion transport, and transcriptional regulation, suggest a multi-pronged approach to maintaining cellular homeostasis and mitigating the effects of stress on gill function. In the intestines, the black module displayed the strongest positive correlation with the combined stress, with hub genes such as *ckba*, *smoc1*, and *dpm2* potentially playing key roles in the intestinal response to dual stress. The involvement of these genes in energy metabolism, cellular signaling, and immune function suggests that the intestine activates diverse mechanisms to cope with stress conditions. In the liver, the brown and turquoise modules showed strong positive correlations with dual stress, while the yellow module exhibited a strong negative correlation. Hub genes such as *golga4*, *arf1*, and *copa* were identified as potentially important regulators in the liver. These genes are involved in vesicle trafficking, Golgi apparatus function, and protein transport, suggesting that dual stress significantly affects protein processing and transport within the liver.

### 4.4. Endoplasmic Reticulum Stress and Immune System Activation

Our functional enrichment analysis further underscored the role of ER stress in the dual stress response. The significant enrichment of the “protein processing in the endoplasmic reticulum” pathway across all three tissues, particularly in the liver, strongly implicates ER stress as a central component of SK’s response to combined stressors. The upregulation of genes involved in various aspects of the pathway, including protein chaperone recognition (e.g., *nef*, *bip*, *grp94*, *hsp40*), protein folding (e.g., *crt*, *cnx*, *pdis*, *erp57*), protein export (i.e., *ergic53*, *vip36*, *sec12*, *sec23/24*, *sec13/31* and *sec61*), and ERAD, reflects a coordinated effort to alleviate ER stress and maintain protein homeostasis [53,54]. The upregulation of apoptosis-related genes (e.g., *bak/bax*, *atf4*) and downregulation of the anti-apoptotic *bcl-2* gene in the intestines and liver suggest that prolonged or severe dual stress may overwhelm these protective mechanisms, leading to the activation of apoptotic pathways [55]. This finding contrasts with some studies in other fish species where *bcl-2* is upregulated under stress [56,57], highlighting the potential for species-specific variations in the apoptotic response. This warrants further investigation to determine the precise role of apoptosis in the dual stress response of SK and to explore the potential implications for tissue damage and overall fish health.

Beyond ER stress, our results point to a crucial role of the immune system in SK’s response to dual stress. DEGs in the gills and intestines were significantly enriched in GO terms related to immune response and stimulus response, suggesting activation of innate immune defenses at the interface with the external environment. These tissues, in direct contact with the surrounding water, experience the immediate effects of salinity and temperature fluctuations. The observed downregulation of *cirbp*, a cold-inducible RNA-binding protein often associated with inflammation and cancer [58,59], may indicate dysregulation or suppression of the inflammatory response under dual stress. Furthermore, the differential expression of genes involved in immune cell regulation and signaling, such as *clec4e*, underscores the complex interplay between environmental stress and immune system modulation. These findings highlight the importance of investigating the long-term impacts of combined stress on immune competence and disease susceptibility in SK rainbow trout.

## 5. Conclusions

This study, encompassing 72 RNA-seq datasets derived from gill, intestine, and liver tissues, investigated the molecular responses of “Shuike No. 1” (SK) rainbow trout to salinity (30‰) and temperature (25 °C) stress. Our findings reveal a complex, tissue-specific response to these environmental challenges, with a pronounced synergistic effect observed under dual stress. Differential gene expression analysis highlighted a greater number of DEGs under combined stress compared to individual stressors, underscoring the non-additive nature of the dual stress response. Notably, SK exhibited a robust response to high salinity alone, suggesting a degree of pre-adaptation to osmotic stress. Three genes, *klf9*, *fkbp5a*, and *fkbp5b*, were consistently differentially expressed across tissues and stress conditions, emerging as potential biomarkers for assessing SK’s adaptability to environmental change. Functional enrichment analysis revealed a significant role for ER stress in the dual stress response, particularly the pathway “protein processing in endoplasmic reticulum.” WGCNA identified stress-associated modules and key hub genes in each tissue. These hub genes were primarily involved in gene expression, ER function, disease immunity, energy metabolism, and substance transport. Collectively, our results indicate that high temperature exerts a more pronounced effect on SK than high salinity, with a significant synergistic effect under combined stress. The identification of key genes, pathways, and modules associated with the dual stress response provides a valuable foundation for future breeding programs aimed at enhancing stress tolerance and promoting the sustainable aquaculture of SK rainbow trout in the face of global warming.

## Data Availability

The original contributions presented in this study are included in the article/Supplementary Materials. Further inquiries can be directed to the corresponding authors.

## Supplementary Materials

The following supporting information can be downloaded at: https://www.mdpi.com/article/10.3390/biology14010049/s1,Figure S1: Scale independence and mean connectivity for weighted gene co-expression network analysis (WGCNA) in the three tissues of rainbow trout “Shuike No. 1” (SK). (A) Gill. (B) Intestine. (C) Liver; Figure S2: Functional enrichment analysis for genes of target modules in rainbow trout (SK) gill, intestine, and liver tissues. (A) Some significantly enriched GO terms. (B) Some significantly enriched KEGG pathways; Figure S3: Identification of gene modules associated with dual salinity and temperature stress in the intestine. (A) Cluster dendrogram of the top 5000 genes with the most variation in the 18 RNA-seq datasets of the intestines. These genes were clustered into 15 distinctly independent modules, which were marked with various colors. (B) Eigengene adjacency tree and heatmap, illustrating the hierarchical clustering of modules based on their eigengene adjacency, revealing distinct expression patterns. (C) Topological Overlap Matrix (TOM) plot. Four hundred genes were randomly selected from the 5000 most variable genes for visualization. Red color intensity represents the degree of topological overlap between genes (deeper red indicates a higher overlap). (D) Heatmap depicting the correlations between module eigengenes and high salinity and/or high temperature. Negative and positive correlations are indicated in blue and red, respectively; Figure S4: Identifying hub genes associated with dual stress in the intestine. (A, B, and C) Scatter plots of gene significance (GS) and module membership (MM) for the black (A), pink (B), and green-yellow (C) modules. Genes with GS > 0.5 and MM > 0.5 (indicated by horizontal lines) are potential hub genes, highlighted in darker colors. (D, E, and F) Co-expression networks of candidate genes in the black (D), pink (E), and green-yellow (F) modules, constructed based on intramodular connectivity. Circle size is proportional to the intramodular connectivity. Genes with connectivity greater than 43.03 (D), 141.33 (E), and 26.05 (F) are potential hub genes. (G) Venn diagram of hub genes in the black, pink, and green-yellow modules. (H) Clustering heatmap of hub gene expression in the black, pink, and green-yellow modules. Expression levels were normalized using log_2_ (FPKM + 1); Figure S5: Identification of gene modules associated with dual salinity and temperature stress in the liver. (A) Cluster dendrogram of the top 5000 genes with the most variation in the 18 RNA-seq datasets of the liver. These genes were clustered into nine distinctly independent modules, which were marked with various colors. (B) Eigengene adjacency tree and heatmap, illustrating the hierarchical clustering of modules based on their eigengene adjacency, revealing distinct expression patterns. (C) Topological Overlap Matrix (TOM) plot. Four hundred genes were randomly selected from the 5000 most variable genes for visualization. Red color intensity represents the degree of topological overlap between genes (deeper red indicates a higher overlap). (D) Heatmap depicting the correlations between module eigengenes and high salinity and/or high temperature. Negative and positive correlations are indicated in blue and red, respectively; Figure S6: Identifying hub genes associated with dual stress in the liver. (A, B, and C) Scatter plots of gene significance (GS) and module membership (MM) for the brown (A), turquoise (B), and yellow (C) modules. Genes with GS > 0.6 and MM > 0.6 (indicated by horizontal lines) are potential hub genes, highlighted in darker colors. (D, E, and F) Co-expression networks of candidate genes in the brown (D), turquoise (E), and yellow (F) modules, constructed based on intramodular connectivity. Circle size is proportional to the intramodular connectivity. Genes with connectivity greater than 168.51 (D), 307.88 (E), and 344.03 (F) are potential hub genes. (G) Ven diagram of hub genes in the brown, turquoise, and yellow modules. (H) Clustering heatmap of hub gene expression in the brown, turquoise, and yellow modules. Expression levels were normalized using log_2_ (FPKM + 1); Figure S7: A qRT-PCR validation for RNA-seq analysis. A total of 12 genes were selected from DEGs for qRT-PCR and *β-actin* was used as the reference gene for normalization. The results of qRT-PCR and RNA-seq were shown as mean ± SD. The relative expression levels were calculated using the method of 2^−ΔΔCt^ and relative FPKM; Table S1: Gene-specific primers for qRT-PCR validation experiment; Table S2: Statistics of RNA-seq datasets from three tissues of rainbow trout “Shuike No. 1” (SK) at four different culture conditions; Table S3: The number of differentially expressed genes (DEGs) under high salinity and/or high temperature stress in three tissues; Table S4: Common DEGs detected in all the pairwise comparisons; Table S5: Gene number of various modules in WGCNA for three tissues of rainbow trout (SK); Table S6: Hub genes associated with rainbow trout (SK) under dual stress of salinity and temperature; Table S7: Hub genes were significantly enriched GO terms and KEGG pathways.
