# Supplementary figures and images for: Transcriptomic Insights into Dual Temperature–Salinity Stress Response in “Shuike No. 1”, a Pioneering Rainbow Trout Strain Bred in China
Source: Biology (Basel). 2025 Jan 10;14(1):49. doi: 10.3390/biology14010049 (PMC11761190; doi:10.3390/biology14010049)

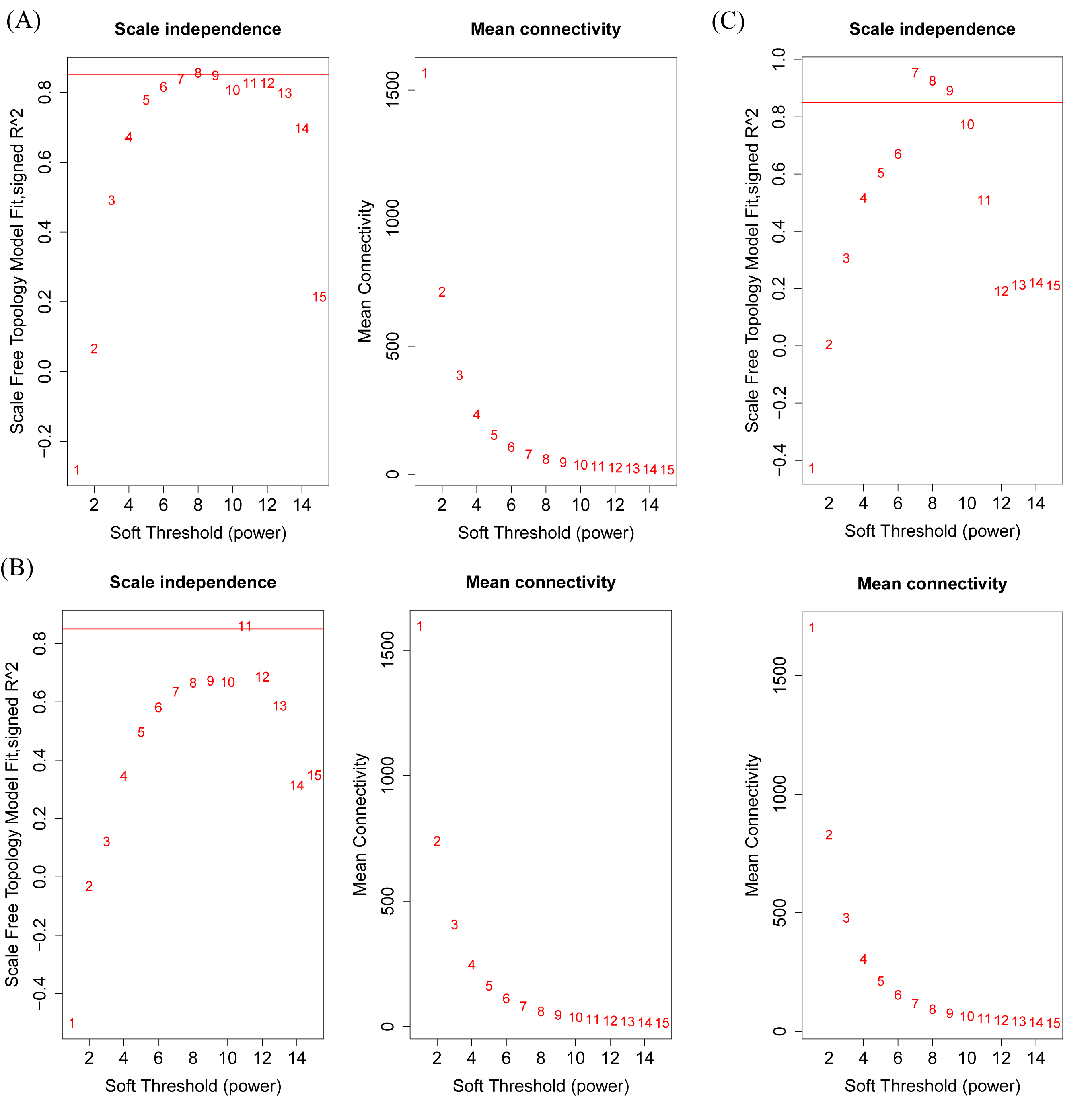

Supplement: Supplementary file 1 [file biology-14-00049-s001.zip › Figure S1.tif]

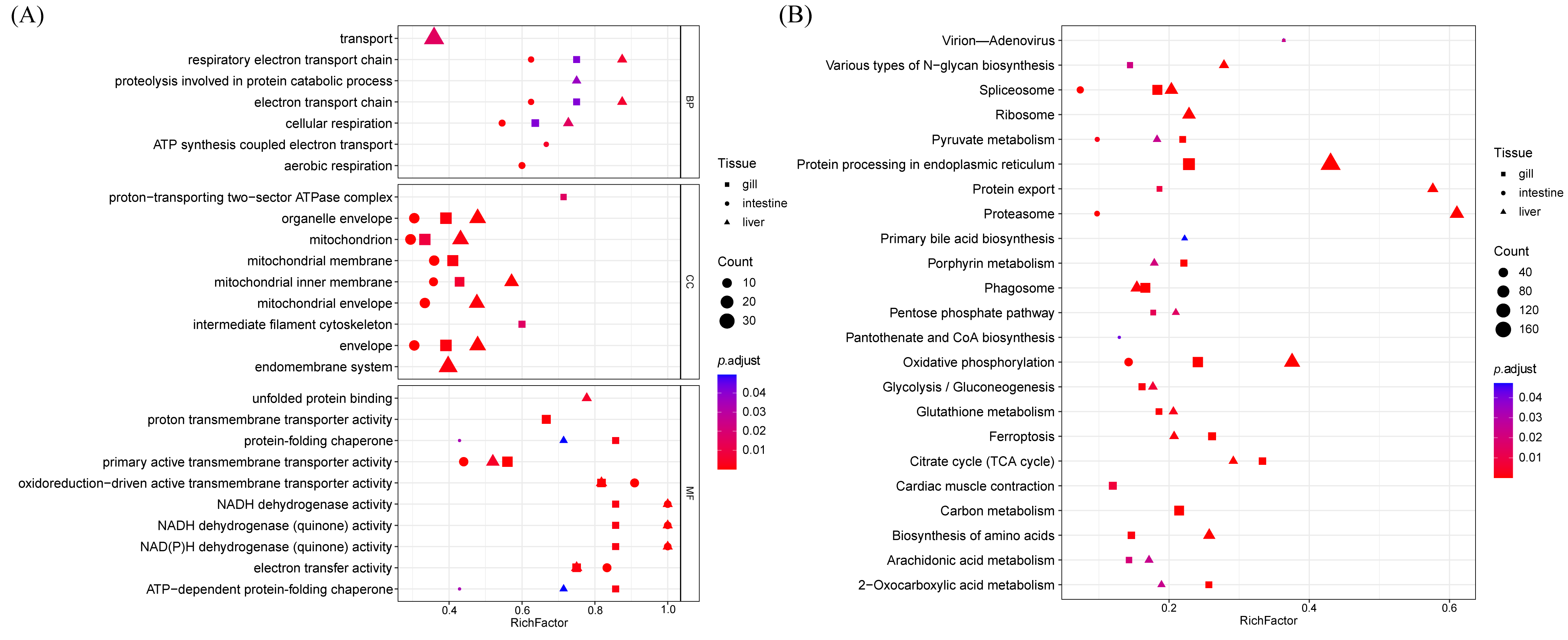

Supplement: Supplementary file 1 [file biology-14-00049-s001.zip › Figure S2.tif]

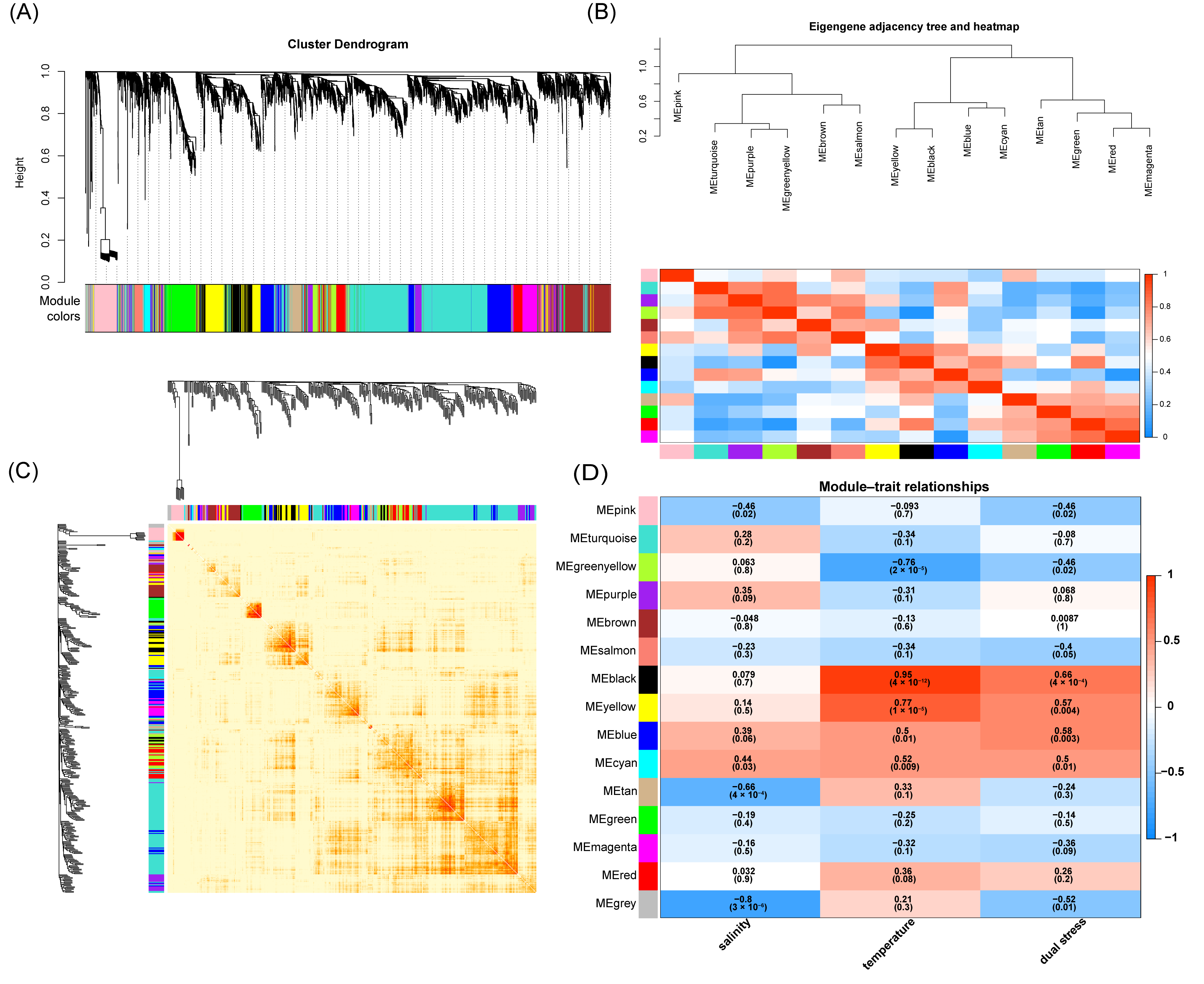

Supplement: Supplementary file 1 [file biology-14-00049-s001.zip › Figure S3.tif]

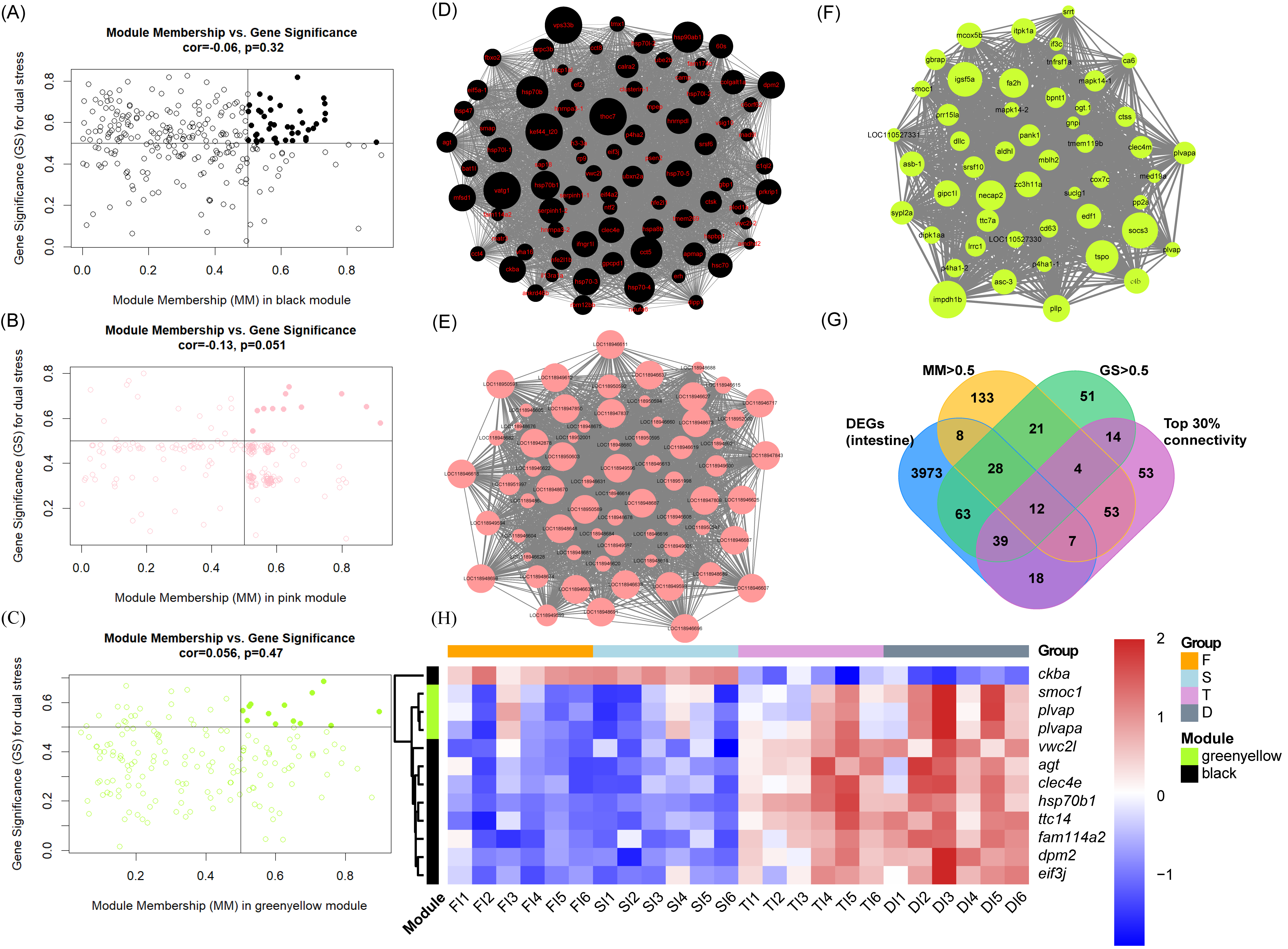

Supplement: Supplementary file 1 [file biology-14-00049-s001.zip › Figure S4.tif]

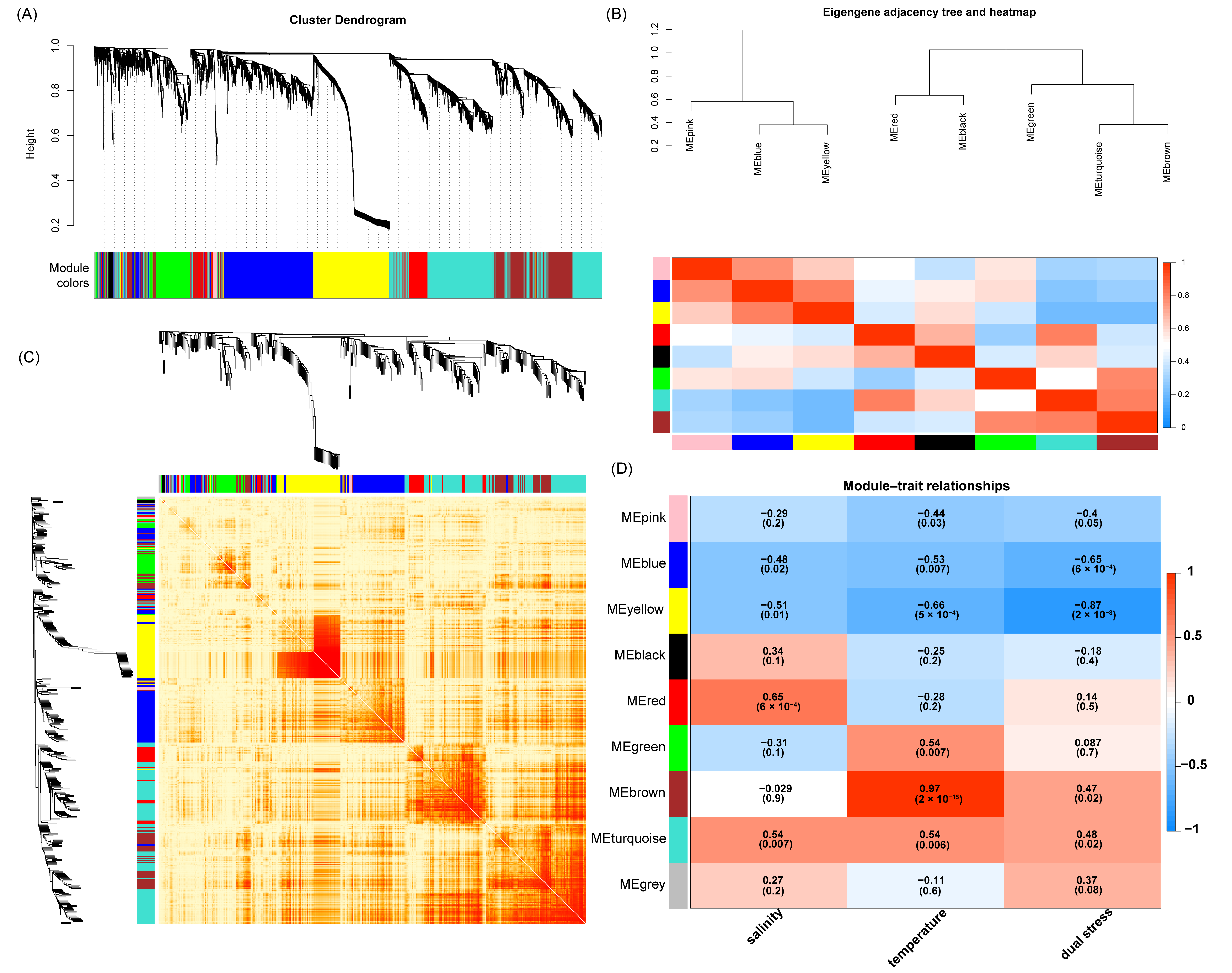

Supplement: Supplementary file 1 [file biology-14-00049-s001.zip › Figure S5.tif]

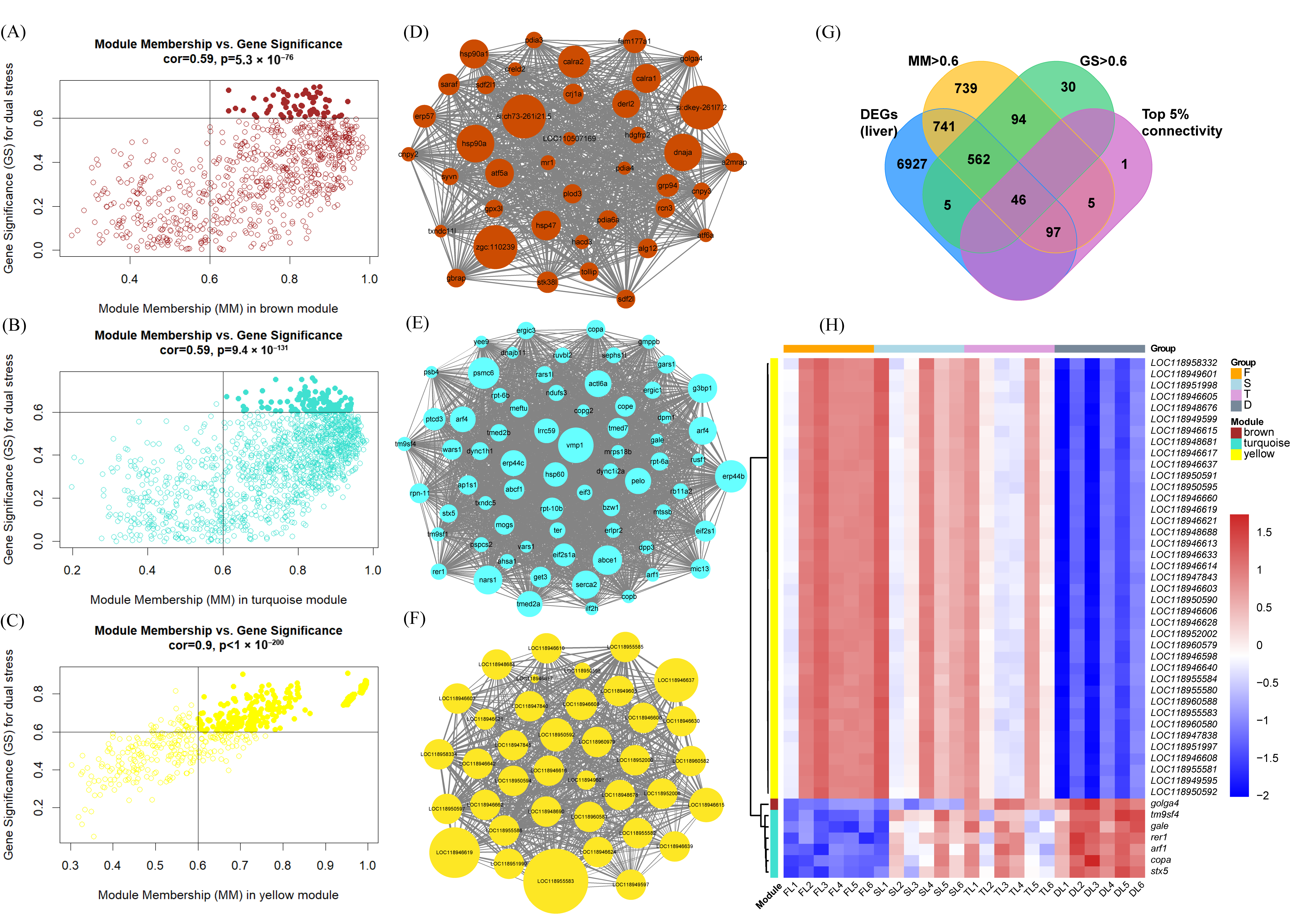

Supplement: Supplementary file 1 [file biology-14-00049-s001.zip › Figure S6.tif]

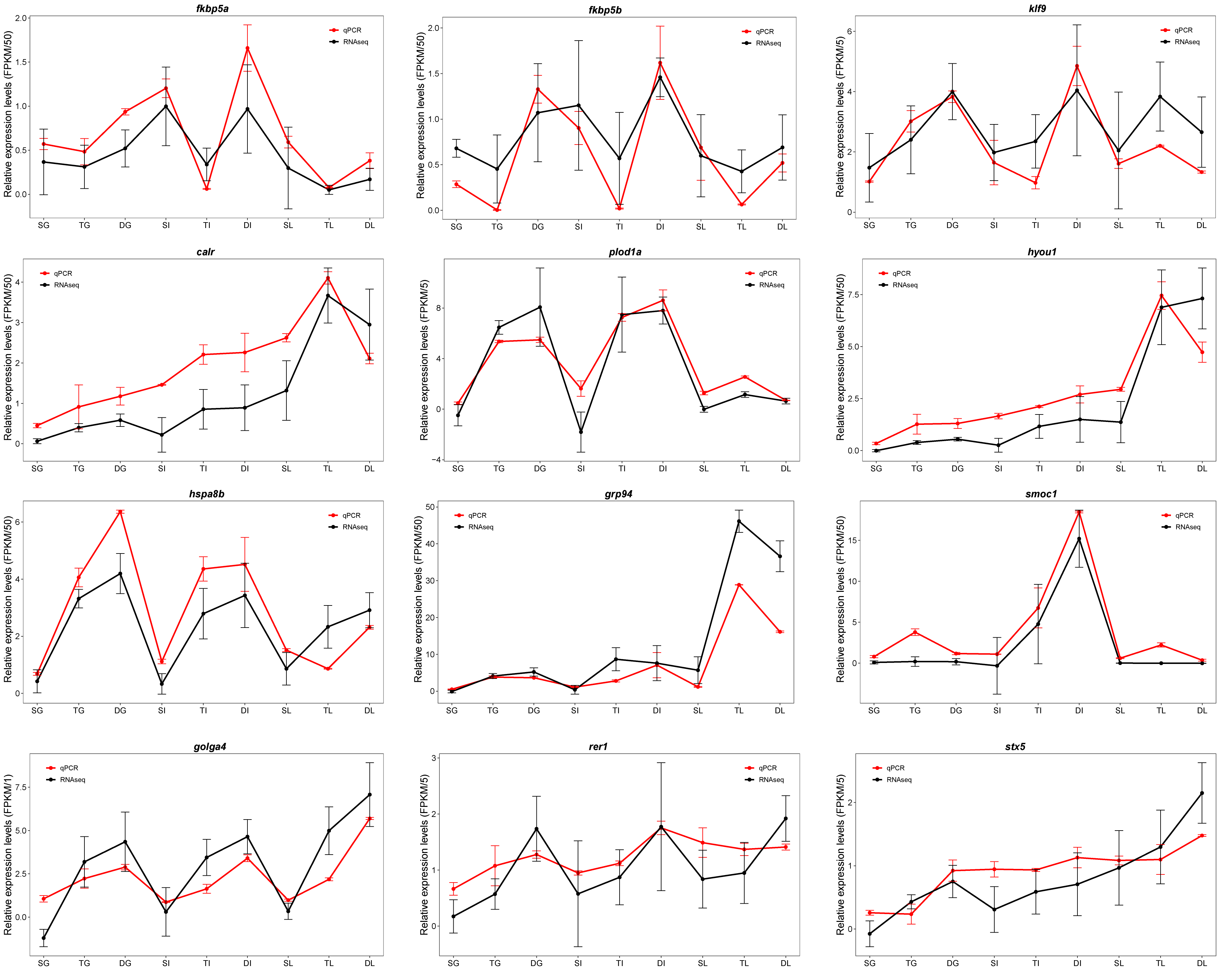

Supplement: Supplementary file 1 [file biology-14-00049-s001.zip › Figure S7.tif]
